# Observational Study of Early Outcomes in Single-Vessel and Multiple-Vessel Renal Allograft

**DOI:** 10.7759/cureus.27579

**Published:** 2022-08-01

**Authors:** Mukteshwar Kumar, Ishwar Ram Dhayal

**Affiliations:** 1 Urology and Renal Transplant, Dr. Ram Manohar Lohia Institute of Medical Sciences, Lucknow, IND

**Keywords:** vascularity, anastomoses, serum creatinine, cit, wit, donors, multiple vessel, allograft, esrd, renal transplantation

## Abstract

Introduction

End-stage renal disease (ESRD) is a global problem with a similar worldwide need for renal replacement therapy. However, the availability of transplant organs remains a challenge. Therefore, we conducted this study to measure early outcomes (up to one month) of renal allograft functions after renal transplant by assessing graft vascularity. We also measured the changes in serum creatinine and hemoglobin levels in single- and multiple-vessel renal allograft recipients.

Methods

We conducted an observational study of 108 renal allograft patients for two years. The study included all renal allograft recipients during the study period. The study excluded patients with a history of renal transplant, patients on antithrombotic therapy, a donor with previous surgery of graft kidney, and patients with anatomic abnormalities. Eighty-five patients were in the single-vessel group, and 23 were in the multiple-vessel group. Donors and recipients received standard pretransplant workup, including donor CT angiography and human leukocyte antigen crossmatching. We performed laparoscopic donor nephrectomies for all participants and perfused all renal arteries separately with Renograph solution (Claris Lifesciences, North Brunswick, NJ). A renal transplant was done in the right or left iliac fossa, noting warm and cold ischemia times. In single-vessel recipients, we anastomosed the renal artery to the external iliac artery (EIA), the renal vein to the external iliac vein (EIV), and the ureter to the bladder via a modified Lich-Gregoir technique. In multiple-vessel recipients, we performed bench anastomosis to make a single vessel, or we anastomosed vessels separately to the EIA, EIV, or the inferior epigastric artery for patients with a second small renal artery. We measured postoperative serum creatinine and hemoglobin levels for one month. In addition, we assessed graft vascularity with ultrasound-guided (USG) Doppler in the first postoperative week. We used IBM SPSS Statistics for Windows, version 21.0. (IBM Corp., Armonk, NY) for all data analyses.

Results

Warm ischemia time (WIT) was longer in multiple-vessel patients than in single-vessel patients, but the difference was insignificant (p=0.054). Cold ischemia time (CIT) was significantly longer in the multiple-vessel group than in the single-vessel group (p=0.048). We found no significant difference in serum creatinine or hemoglobin levels between groups during the study period. Perigraft collection occurred in three single-vessel patients and decreased vascularity in two multiple-vessel patients, according to USG Doppler.

Conclusions

We conducted this study to measure early outcomes of renal allograft functions after renal transplant by assessing graft vascularity, serum creatinine, and hemoglobin levels in single- and multiple-vessel renal allograft patients. According to our results, renal transplantation is not inferior in multiple-vessel allograft patients. We found no significant difference in serum creatinine levels one month postoperatively. Using multiple-vessel donors helps increase the limited donor pool, which is ultimately better for managing ESRD patients.

## Introduction

Unfortunately, only half of the end-stage renal disease (ESRD) patients worldwide receive renal replacement therapy, while the other half die [1]. Kidney transplantation is the optimal treatment of ESRD because it provides recipients with the best quality of life [2]. Live kidney donation increases with multiple vessels to meet the current demand and increase the donor pool. Previously, using kidneys with multiple vessels was discouraged because of complex bench anastomosis, prolonged ischemia time, and poor transplant outcomes in the recipients [3]. Multiple renal arteries are found in 18%-30% unilaterally and 10% bilaterally [4]. Renal transplantation with multiple arteries carries a significant index of vascular and urological complications [5]. Now, vascular surgical techniques intra or ex vivo used for anastomosis in cases of multiple vessels are achieving results similar to those with single vessels [4,5]. We conducted this study to measure renal allograft function up to one month after transplant, assessing the changes in serum creatinine and hemoglobin levels. We also assessed graft vascularity using ultrasound-guided (USG) Doppler in single-vessel and multiple-vessel graft recipients.

## Materials and methods

We conducted a two-year observational study of 108 renal allograft recipient patients. All renal allograft recipients were included in the study during the two years. The study excluded patients with a history of renal transplant, patients on antithrombotic therapy, donors with previous surgery of graft kidney, and patients with anatomic abnormalities (e.g., double ureter). The study population comprised 85 patients in the single-vessel group and 23 patients in the multiple-vessel group. 

We performed standard pretransplant workups for donors and recipients, including donor CT angiography and human leukocyte antigen crossmatching. Laparoscopic donor nephrectomy was performed in all cases. All renal arteries were perfused separately with Renograph solution (Claris Lifesciences, North Brunswick, NJ). A renal transplant was done in the right or left iliac fossa, and we recorded warm ischemia time (WIT) and cold ischemia time (CIT). In single-vessel recipients, a single renal artery was anastomosed to the external iliac artery (EIA), the renal vein to the external iliac vein (EIV), and the ureter to the bladder using a modified Lich-Gregoir technique. In the case of multiple vessels, we performed bench anastomosis to make a single vessel, or we anastomosed vessels separately to the EIA, EIV, or inferior epigastric artery (IEA) if patients had a second small renal artery. We recorded postoperative changes in serum creatinine and hemoglobin levels for one month. Graft vascularity was assessed via USG Doppler in the first postoperative week. We also noted any complications like perinephric collection and decreased vascularity. The study was approved by the Institutional Ethics Committee (IEC No. 48/19), and the study does not affect the primary treatment protocol. There were no adverse effects due to the results of this study.

Statistical analysis

The results are presented in frequencies, percentages, and mean ± SD. The unpaired t-test was used to compare continuous variables between the groups. We used the paired t-test to compare the mean change in various parameters within the groups before and after surgery. P <0.05 was considered significant. The analysis was carried out using IBM SPSS Statistics for Windows, version 21.0. (IBM Corp., Armonk, NY).

## Results

Of the 108 patients included, 99 (91.7%) were male, and nine (8.3%) were female. Most patients (44.4%) were aged 21-30 years, while 28.7% of patients were aged 31-40 years. Basiliximab was used in 51 patients (47.22%), antithymocyte globulin in 42 patients (38.8%), Grafalon (Neovii Pharmaceuticals, Rapperswil, Switzerland) in eight patients (7.40%), and seven patients had no induction. The left side kidney was taken in 87 patients (80.55%), while the right kidney was used for donation in 21 patients (19.44%). Eighty-five patients had a single vessel (78.7%), and multiple vessels were present in 23 patients (21.29%). Of these 23 patients, 19 (82.60%) had multiple renal arteries, and four had multiple renal veins (17.4%) (Table 1).

**Table 1:** Distribution of anastomosis among multiple vessels. EIA: External iliac artery; IEA: Inferior epigastric artery; EIV: External iliac vein; MRA/V: Multiple renal artery/vein.

Anastomosis	Multiple vessel (n = 23)	
	MRA	MRV
EIA	13 (56.52%)	0
EIA, IEA	6 (26.08%)	0
EIV	0	4 (17.4%)
Total	19 (82.60%)	4 (17.4%)

Among multiple-vessel patients, two (50%) had a single vein formed on the bench and anastomosed to the EIV. Another two patients’ veins were anastomosed separately to the EIV. Of 19 patients with multiple renal arteries, three (15.78%) arteries were joined on the bench to make a single vessel that was anastomosed to the EIA. In 10 patients (52.63%), renal arteries were anastomosed separately to the EIA. In six patients (31.57%), the main renal artery was anastomosed to the EIA, and the second small artery was anastomosed to the IEA (Table 2).

**Table 2:** Distribution of multiple arterial anastomosis. EIA: External iliac artery; IEA: Inferior epigastric artery.

Status	Number of patients (n = 19)	Anastomosis
Multiple arteries joined as single vessel, anastomosis to EIA	3 (15.78%)	EIA
Multiple arteries anastomosis to EIA	10 (52.63%)	EIA
Multiple arteries anastomosis to EIA, IEA	6 (31.57%)	EIA, IEA
Total	19 (100%)	-

**Figure 1:**
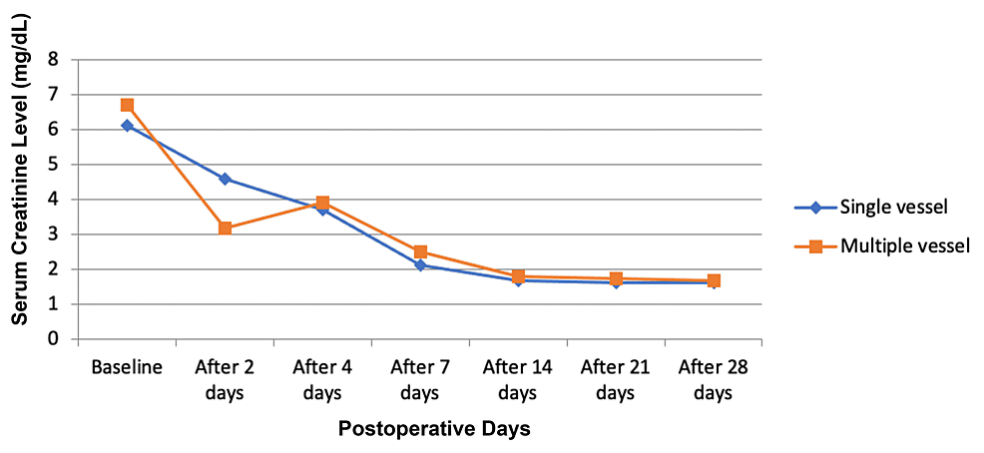
Comparison of serum creatinine level among single- and multiple-vessel patients.

The mean WIT in the single-vessel group was 5.95 ± 2.68 minutes, and the mean WIT in the multiple-vessel group was 6.56 ± 2.27 minutes, which was longer but not significantly so. The CIT in the multiple-vessel group was 57.47 ± 16.58 minutes and 52.88 ± 18.49 minutes (p=0.048) in the single-vessel group. Serum creatinine levels showed no significant changes one month postoperatively in both groups except on day 2 when a significant decline was seen in the multiple-vessel group (3.16 ± 2.1 mg/dL) compared to the single-vessel group (4.57 ± 2.8 mg/dL; p=0.046) (Table 3, Figure 1).

**Table 3:** Postoperative comparison of serum creatinine levels in single- and multiple-vessel patients.

Parameter	Mean Serum Creatinine (mg/dL) ± SD		P-value
	Single vessel	Multiple vessel	
Baseline	6.1 ± 4.2	6.69 ± 3.9	0.124
After 2 days	4.57 ± 2.8	3.16 ± 2.1	0.046
After 4 days	3.7 ± 1.1	3.9 ± 1.9	0.221
After 7 days	2.1 ± 0.7	2.5 ± 0.68	0.201
After 14 days	1.66 ± 0.8	1.78 ± 0.56	0.215
After 21 days	1.61 ± 0.7	1.72 ± 0.44	0.111
After 28 days	1.60 ± 0.65	1.66 ± 0.46	0.189

There was no significant difference in mean urine output between the two groups for up to one month. Similarly, we observed no significant difference in drain output between the two groups until seven to 10 days because most of the patients' drains were removed before seven days. Hemoglobin levels declined significantly in the multiple-vessel group on day 7 (7.35 ± 2.47 g/dL) compared to the single-vessel group (8.23 ± 1.87 g/dL), but we found no significant change during the study period. USG Doppler findings were unremarkable in most patients in both groups (80 single-vessel patients, 94.1%; 20 multiple-vessel patients; 86.9%). Perinephric collection with normal vascularity was encountered in three patients (3.52%) in the single-vessel group and one patient (4.34%) in the multiple-vessel group. USG-guided percutaneous nephrostomy was performed on one patient in the single-vessel group. Lack of blood flow in the graft kidney was observed on Doppler in one patient (1.17%) in the single-vessel group, and that patient received a graft nephrectomy. We noted reduced vascularity in two patients (8.7%) in the multiple-vessel group but no decreased vascularity in the single-vessel group (Table 4).

**Table 4:** Comparison of USG Doppler among single and multiple-vessel groups at follow-up duration. USG: Ultrasound-guided; PCN: Percutaneous nephrostomy; MRA: Magnetic resonance angiography; RI: Resistive index.

Parameter	Single vessel	Multiple vessel	P-value
Normal	80 (94.1%)	20 (86.9%)	0.121
Perigraft collection, normal vascularity	3 (3.52%)	1 (4.34%)	0.201
USG PCN done	1 (1.17%)	0 (0%)	0.098
Absent flow in graft kidney, graft nephrectomy	1 (1.17%)	0 (0%)	0.088
Decreased vascularity (MRA>200 cm/sec, RI>0.8)	0 (0%)	2 (8.7%)	0.054
Total	85 (100%)	23 (100%)	-

## Discussion

Renal grafting with multiple arteries carries a high theoretical risk of surgical complications to the living donor and recipient. Osman Y et al. reported that multiple renal arteries were associated with a high rate of posttransplant vascular complications [6]. Han D et al. also concluded that grafts with multiple renal arteries had a higher rate of vascular complications than single arterial grafts [7]. Given the growing incidence of ESRD worldwide, the need for transplant tissue is also growing. This study aimed to measure early outcomes (up to one month) of renal allograft functions after renal transplant by assessing graft vascularity. We also measured changes in serum creatinine and hemoglobin levels in single- and multiple-vessel renal allograft recipients. Our one-month study found no significant difference in graft vascularity or serum creatinine when comparing multiple- and single-vessel patients. 

Our study demographics of primarily male patients aged 21-40 years are similar to those in two similar previous studies [8,9]. Natsis K et al. reported no statistically significant relationship between sex and the location of the supernumerary renal artery [10]. However, Hung CJ et al. [11] reported a higher incidence of the supernumerary renal artery in males than females. Sezer TO et al. concluded the opposite [12]. 

In our study, most patients had single vessels, with a smaller group possessing multiple vessels. Novick et al. reported a similar proportion of single-to-multiple-vessel patients in their study. In our study, 87 patients (80.55%) donated a left kidney, the side that has significant consequences for the arterial anatomy of the living kidney donor available. 

In our study, WIT was longer in multiple-vessel patients than the single-vessel patients, but the difference was insignificant (p=0.054). CIT was significantly longer in the multiple-vessel group than in the single-vessel group (p=0.048). Chabchoub K et al. reported that WIT was significantly longer in their study in the multiple renal arteries group with no influence on the incidence of acute tubular necrosis [13]. 

We found a significantly more significant decline in hemoglobin on day 7 in the multiple-vessel group patients than in single-vessel patients. However, there was no significant difference in hemoglobin one month postoperatively. There was also no significant difference in mean urine output and drain output between groups. These results match those reported by Desai MR et al. [14] and Bakirtas H et al. [15]. Mazzucchi E et al. concluded that there was no significant difference in the occurrence of vascular and urologic complications and delayed graft function between single- and multiple-artery patients [8]. Patients with a vascular velocity >200 cm/second on magnetic resonance angiography [8,16] and a resistive index >0.8 were considered to have decreased vascularity. We noted decreased vascularity in two patients (8.7%) in the multiple vessels group but none in the single-vessel group. 

Our study was limited by its small sample size and short-term follow-up period. Therefore, a more extensive study with a more extended follow-up period is warranted to support our results further.

## Conclusions

Kidney transplantation using grafts with multiple renal arteries is as safe as using grafts with a single renal artery regarding vascular and urological complications and patient and graft survival. Several techniques for bench or in situ reconstructions of multiple renal arteries can reduce the incidence of these vascular complications. Although WIT and CIT were higher in the multiple vessels group, this did not negatively affect early graft function. By using various surgical techniques on the bench, transplantation in multiple-vessel patients is viable and can increase the donor pool for the betterment of ESRD patients.
